# NFE2L1 as a central regulator of proteostasis in neurodegenerative diseases: interplay with autophagy, ferroptosis, and the proteasome

**DOI:** 10.3389/fnmol.2025.1551571

**Published:** 2025-05-01

**Authors:** Hossein Khodadadi, Kamila Łuczyńska, Dawid Winiarczyk, Paweł Leszczyński, Hiroaki Taniguchi

**Affiliations:** ^1^Department of Experimental Embryology, Institute of Genetics and Animal Biotechnology of the Polish Academy of Sciences, Jastrzebiec, Poland; ^2^The Second Department of Psychiatry, Institute of Psychiatry and Neurology in Warsaw, Warsaw, Poland; ^3^Department of Stem Cell Bioengineering Mossakowski Medical Research Institute, Polish Academy of Sciences, Warsaw, Poland; ^4^African Genome Center, University Mohammed VI Polytechnic (UM6P), Ben Guerir, Morocco

**Keywords:** NFE2L1, proteostasis, neurodegenerative diseases, autophagy, ferroptosis, proteasome, oxidative stress, mitochondrial health

## Abstract

Maintaining proteostasis is critical for neuronal health, with its disruption underpinning the progression of neurodegenerative diseases such as Alzheimer’s, Parkinson’s, and Huntington’s diseases. Nuclear Factor Erythroid 2-Related Factor 1 (NFE2L1) has emerged as a key regulator of proteostasis, integrating proteasome function, autophagy, and ferroptosis to counteract oxidative stress and protein misfolding. This review synthesizes current knowledge on the role of NFE2L1 in maintaining neuronal homeostasis, focusing on its mechanisms for mitigating proteotoxic stress and supporting cellular health, offering protection against neurodegeneration. Furthermore, we discuss the pathological implications of NFE2L1 dysfunction and explore its potential as a therapeutic target. By highlighting gaps in the current understanding and presenting future research directions, this review aims to elucidate NFE2L1’s role in advancing treatment strategies for neurodegenerative diseases.

## 1 Introduction: the challenge of maintaining proteostasis in the brain

Proteostasis, or protein homeostasis, is the critical process by which cells maintain the balance between protein synthesis, folding, trafficking, and degradation. This process is vital for cellular integrity and function, especially in neurons, which have to ensure correct synthesis-degradation protein cycle over long lifespans (Hipp et al., 2019). The central nervous system (CNS) is particularly vulnerable to proteostasis disruptions due to neurons’ high metabolic demands and limited regenerative capacity (Hetz and Saxena, 2017). Post-mitotic neurons cannot regenerate, making them highly reliant on robust proteostasis mechanisms for protein quality control. Any failure in these mechanisms can lead to the accumulation of damaged or misfolded proteins, directly contributing to the pathogenesis of neurodegenerative diseases, such as Alzheimer’s (AD), Parkinson’s (PD), Amyotrophic lateral sclerosis (ALS), and Huntington’s disease (HD) (Morrone et al., 2023). The brain’s high energy demands and reliance on mitochondrial ATP production further complicate proteostasis maintenance. Reactive oxygen species (ROS), a byproduct of mitochondrial activity, exacerbate neuronal vulnerability by damaging proteins, lipids, and nucleic acids (Türker et al., 2021; Glick et al., 2010). Moreover, the brain’s inability to generate new cells in response to damage, unlike many other tissues, makes neurons heavily reliant on proteostasis to mitigate the harmful effects of oxidative stress. Therefore, enhancing the brain’s ability to preserve proteostasis under oxidative stress is a key therapeutic strategy for mitigating neurodegeneration (Stavoe and Holzbaur, 2019).

Several key pathways regulate neuronal proteostasis, including the proteasome system (UPS), autophagy, and ferroptosis. The UPS is responsible for the targeted degradation of short-lived or damaged proteins and plays a central role in maintaining cellular health by preventing the accumulation of toxic protein aggregates (McKinnon and Tabrizi, 2014). This process is tightly regulated, especially under stress conditions when the cell requires enhanced proteasomal activity to clear misfolded proteins (McKinnon and Tabrizi, 2014; Kandel et al., 2024). Autophagy, another essential proteostasis mechanism, complements the UPS by degrading larger protein aggregates, organelles, and damaged structures through lysosomal pathways (Liang and Sigrist, 2018). Dysregulation in autophagy can exacerbate neurodegeneration, as dysfunctional components accumulate, worsening cellular damage and accelerating disease progression (Jiang et al., 2021).

Ferroptosis, a regulated form of programmed cell death, is closely linked to oxidative stress and iron dysregulation, emphasizing the role of lipid peroxidation in neuronal damage (Jiang et al., 2021). Ferroptosis has been implicated in neurodegenerative diseases like AD and PD, where iron accumulation and oxidative stress contribute to neuronal loss (Mahoney-Sánchez et al., 2021; Wang et al., 2022b; Bao et al., 2021). Both the UPS and autophagy play pivotal roles in preventing ferroptosis by maintaining cellular homeostasis, regulating oxidative stress and lipid metabolism. When these systems fail, iron dysregulation and lipid peroxidation can trigger ferroptosis, leading to neurodegeneration (Zhou et al., 2020; Lu et al., 2024). Together, these pathways form an interconnected network essential to neuronal health.

NFE2L1 is a transcription factor that orchestrates proteostasis by regulating these pathways (Liu et al., 2023). By enhancing proteasomal activity, NFE2L1 helps maintain protein homeostasis, preventing the accumulation of toxic protein aggregates that contribute to cellular dysfunction and neurodegenerative diseases (Taniguchi et al., 2017; Chandran et al., 2023; Li et al., 2024). Additionally, NFE2L1 influences other key proteostasis mechanisms, including autophagy (Furuya et al., 2014; Hatanaka et al., 2023) and potentially ferroptosis (Kotschi et al., 2022; Ofoghi et al., 2024), further supporting its role as a central regulator of cellular stress response pathways. Given its involvement in managing proteostasis and mitigating oxidative damage, which accelerates neurodegeneration, NFE2L1 represents a promising target for therapeutic strategies aimed at restoring cellular balance and preventing neurodegenerative progression.

This review explores NFE2L1 as a key regulator of proteostasis in neurodegenerative diseases, highlighting its role in controlling autophagy, ferroptosis, and the proteasome. We will examine the mechanisms by which NFE2L1 governs these critical pathways, highlighting its role in regulating oxidative stress and maintaining protein homeostasis within neurons. By modulating the activity of the proteasome and autophagy, NFE2L1 plays a pivotal role in clearing damaged proteins and organelles, preventing the accumulation of toxic aggregates that contribute to neurodegeneration. Additionally, we will explore how NFE2L1 influences ferroptosis, a form of programmed cell death implicated in neurodegenerative diseases, and the potential therapeutic benefits of targeting this transcription factor to restore proteostasis and protect neuronal function. Ultimately, this review aims to provide a comprehensive understanding of how NFE2L1 functions as a master regulator of proteostasis and its therapeutic potential in treating neurodegenerative disorders.

## 2 Proteostasis mechanisms in neurodegeneration: proteasomal function, autophagy, and ferroptosis

Proteostasis mechanisms represent an intricate network of cellular processes that ensure protein homeostasis, a critical balance particularly vital in non-proliferative neurons (Díaz-Villanueva et al., 2015; Zhu and Cohen, 2023). These cells are highly susceptible to the toxic effects of misfolded or aggregated proteins. The UPS, autophagy, and ferroptosis are key players in proteostasis, each contributing uniquely to cellular health and resilience against neurotoxicity.

### 2.1 The ubiquitin-proteasome system: the first line of defense against protein aggregation

*Key takeaways:*

- *UPS activity is reduced in neurodegenerative disorders*
- *Protein accumulation is a hallmark of neurodegenerative disorders*
- *UPS dysfunction is linked to impaired function in neurons*

The UPS is central to cellular proteostasis, mediating the targeted degradation of damaged or misfolded proteins. Through ubiquitination, proteins are marked for recognition by the proteasome, which subsequently breaks them down into peptides (Kleiger and Mayor, 2014).

In neurodegenerative disorders, a decline in UPS function has been directly linked to the accumulation of toxic protein aggregates that overwhelm cellular defenses and trigger neuronal death (Bendotti et al., 2012; McKinnon and Tabrizi, 2014). In a study conducted by Bendotti et al. (2012), they highlighted the dysfunction of both constitutive and inducible components of the UPS in ALS. The research demonstrated that the accumulation of ubiquitin-positive inclusions in ALS neurons is strongly associated with impaired proteasomal activity. In particular, the study showed that mutant SOD1 in familial ALS models contributes to protein aggregation and motor neuron death due to UPS dysfunction (Bendotti et al., 2012). Additionally, Bendotti et al. (2012) found that the inducible subunits of the proteasome, which form the immunoproteasome, are overexpressed early in ALS progression. This suggests a dual role of UPS dysfunction, not only in protein degradation but also in the modulation of immune responses, with implications for immune-inflammatory reactions in ALS pathology (Bendotti et al., 2012). The examples from this study underscores the complex role of the UPS in ALS and highlights potential therapeutic targets aimed at both proteasomal dysfunction and immune modulation.

Aging further compounds this vulnerability by reducing proteasomal activity, leading to an accumulation of misfolded and damaged proteins, which in turn overwhelms the cellular quality control mechanisms (Tsakiri and Trougakos, 2015). The age-related decline in proteasomal function is particularly detrimental in neurons, given their post-mitotic nature and the long lifespan of neural cells (Gray et al., 2003; McKinnon and Tabrizi, 2014). This reduction in proteasomal capacity contributes to a failure in proteostasis, which is crucial for maintaining neuronal function. As a result, neurons become less capable of managing misfolded proteins, which can aggregate and form toxic species that disrupt cellular functions (McKinnon and Tabrizi, 2014). This phenomenon is particularly prominent in neurodegenerative diseases, where protein aggregates, such as tau in AD (Weng and He, 2021), *α*-synuclein in PD (Ebrahimi-Fakhari et al., 2011), and TDP-43 in ALS (Yin et al., 2021), play a central role in disease pathogenesis.

Experimental models of ALS, AD, and PD demonstrate that enhancing proteasome function through pharmacological agents or genetic modulation can reduce the accumulation of these toxic proteins, mitigating disease progression and improving cellular health. These findings suggest that therapeutic strategies targeting the UPS could offer a promising avenue for alleviating disease pathology by restoring protein degradation capacity. However, the complexity of proteostasis maintenance goes beyond the UPS, as the system interacts with other cellular processes, particularly autophagy.

### 2.2 Autophagy: the cellular recycling system

*Key takeaways:*

- *Autophagy clears organelles and proteins through lysosomal pathways*
- *Autophagy can compensate for proteasome dysfunction*
- *Autophagy dysfunction is often observed in neurodegenerative disorders*

**Figure 1:**
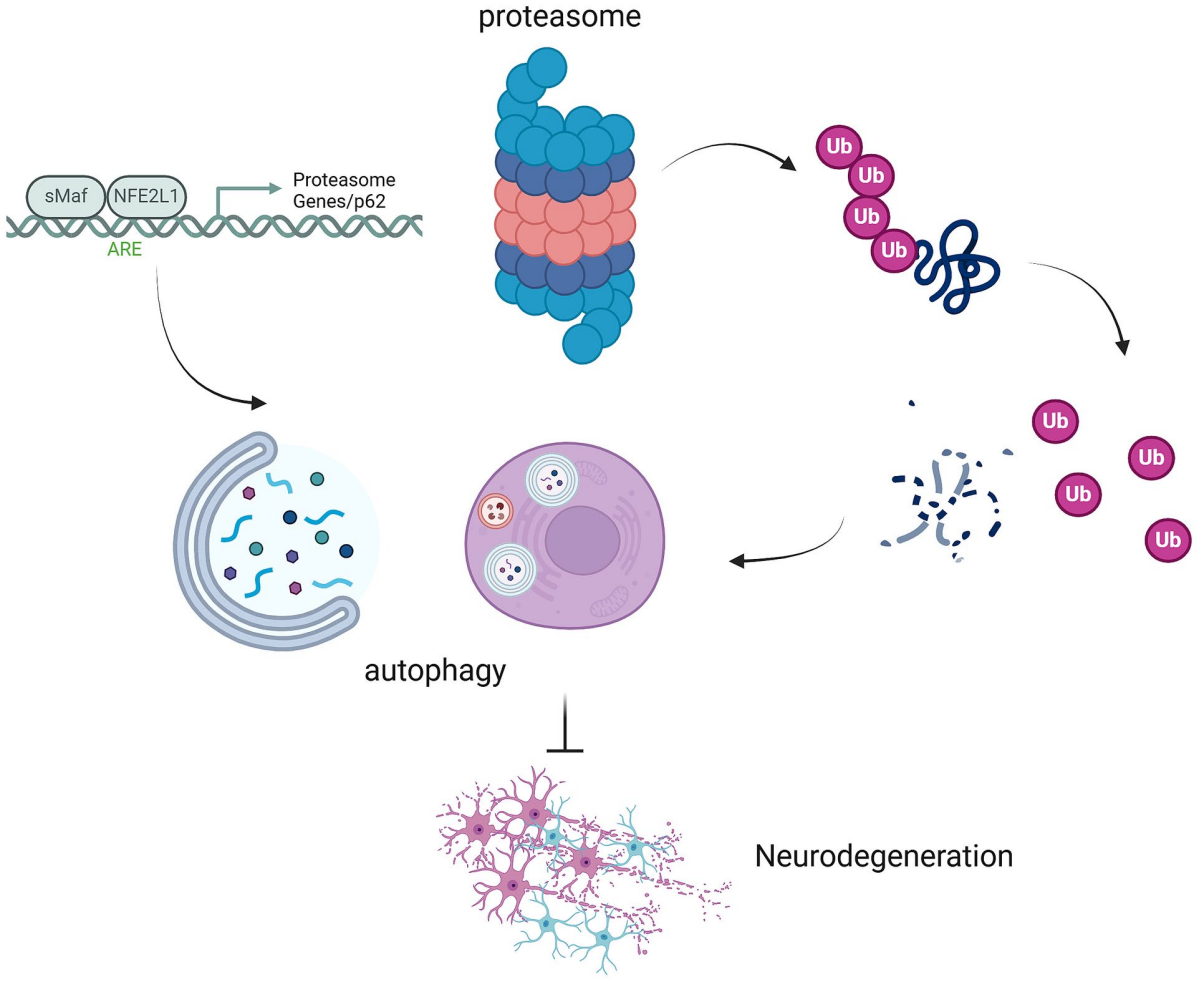
Interplay between proteasomal ubiquitination, autophagy, and neurodegeneration mediated by NFE2L1 and p62. This schematic illustrates the critical interplay between proteasomal ubiquitination, autophagy, and the development of neurodegenerative diseases, highlighting the roles of MAF/NFE2L1, proteasome genes, and p62 in regulating these processes. The proteasome, through the ubiquitination of damaged proteins, and autophagy, which degrades damaged organelles and proteins, work in tandem to maintain cellular proteostasis. NFE2L1 (NRF1), a key regulator of proteostasis, orchestrates the expression of genes involved in the proteasome and autophagic systems. p62 acts as a scaffold protein, linking the proteasome and autophagy pathways by recognizing ubiquitinated proteins for degradation. Disruption in these pathways, due to NFE2L1 dysfunction or p62 aggregation, leads to the accumulation of toxic proteins, contributing to neurodegeneration. This figure illustrates how impaired proteostasis mechanisms in neuronal cells contribute to neurodegenerative diseases such as AD, PD, and ALS.

Autophagy is a fundamental cellular process responsible for degrading damaged organelles and misfolded proteins via lysosomal pathways. It begins with the formation of autophagosomes, which encapsulate cellular debris, followed by their fusion with lysosomes for degradation (Mizushima, 2007). As we age, autophagy efficacy declines, leading to the accumulation of toxic protein aggregates that exacerbate neuronal damage and accelerate disease progression (Aman et al., 2021). Autophagy also works in concert with other proteostatic mechanisms, notably the UPS, to form an integrated cellular defense against stress (Pohl and Dikic, 2019; Kwon and Ciechanover, 2017). When proteasomal degradation is impaired, autophagy often compensates by clearing misfolded proteins. This interplay underscores the interconnected nature of proteostasis pathways (Figure 1).

In neurodegenerative diseases such as AD (Di Meco et al., 2020) and PD (Karabiyik et al., 2017), dysfunction in both the UPS and autophagy is frequently observed, contributing to disease progression (Ghavami et al., 2014). For instance, Tsakiri et al. demonstrated that proteasome dysfunction in Drosophila led to widespread proteome instability, mitochondrial dysfunction, and genomic instability, triggering compensatory autophagic responses (Tsakiri et al., 2019). Similarly, a study by Lin et al. on globoid cell leukodystrophy (GLD) revealed that impairment of both the UPS and autophagy led to the accumulation of ubiquitin and p62 aggregates, particularly in the white matter of the brain and spinal cord, contributing to neuroinflammation and demyelination (Lin et al., 2020). In both studies, inhibition of autophagy or UPS exacerbated cellular damage, highlighting the importance of these pathways in maintaining cellular homeostasis. Together, these findings underscore the critical role of autophagy and proteasome function in protecting against cellular damage.

Therefore, targeting autophagic processes to enhance their function represents a promising therapeutic approach for restoring cellular balance and mitigating neurodegenerative diseases where proteostasis is disrupted.

### 2.3 Ferroptosis: the role of iron and lipid peroxidation in neuronal death

*Key takeaways:*

- *Ferroptosis involves the accumulation of lipid peroxides and ROS*
- *Elevated brain iron levels strongly correlate with cognitive decline*
- *Dysfunction in lipid metabolism can overwhelm autophagic pathways*

Ferroptosis is an iron-dependent cell death and involves the accumulation of lipid peroxides and ROS, compromising membrane integrity (Xie et al., 2016). Elevated iron levels, commonly observed in neurodegenerative diseases such as AD (Ayton et al., 2020) and PD (Hare et al., 2017), amplify susceptibility to ferroptosis, underscoring the importance of iron homeostasis in neuronal health.

Studies reveal that iron’s role in neurodegeneration extends beyond oxidative stress, as it facilitates lipid peroxidation and accelerates disease progression (Masaldan et al., 2019; Jakaria et al., 2021; Ryan et al., 2023). Elevated brain iron levels, detectable via advanced imaging techniques such as quantitative susceptibility mapping (QSM), strongly correlate with cognitive decline, providing evidence for the “iron hypothesis” in neurodegeneration (Apostolakis and Kypraiou, 2017; Masaldan et al., 2019).

Additionally, ferroptosis interacts with proteostatic mechanisms like autophagy. Dysfunction in lipid metabolism or oxidative stress management can overwhelm autophagic pathways, propagating ferroptotic damage (Kang and Tang, 2017). Ferritinophagy, the autophagic degradation of ferritin, releases labile iron, further exacerbating ferroptosis susceptibility (Biasiotto et al., 2016; Tang et al., 2018). This link highlights the interplay between iron storage and the vulnerability of neurons in conditions like AD (An et al., 2024), PD (Boag et al., 2022), and ALS (Bu et al., 2019).

Therapeutic interventions that modulate ferroptosis hold potential for reducing oxidative stress and neuronal loss in neurodegenerative conditions, offering a pathway to restore cellular homeostasis. Key therapeutic strategies include iron chelators (Feng et al., 2022) such as deferiprone (Rayatpour et al., 2022) and deferoxamine (Yao et al., 2019), which have shown efficacy in reducing iron accumulation and mitigating ferroptosis-induced damage in both preclinical and clinical studies (Bruzzese et al., 2023). These treatments not only alleviate oxidative stress but also reduce the progression of motor and cognitive deficits in disorders like PD (Martin-Bastida et al., 2017) and AD (Farr and Xiong, 2020; Fawzi et al., 2020).

Furthermore, enhancing the activity of glutathione peroxidase 4 (GPX4) or boosting cellular glutathione (GSH) levels has been demonstrated to prevent lipid peroxidation, a critical step in ferroptosis (Zhang et al., 2024c). This approach has shown promise in experimental models of AD and PD, as well as other neurological disorders like ALS. Emerging evidence also implicates lipid antioxidants, such as vitamin E (Zhang et al., 2022) and ferrostatin-1 (Ge et al., 2022), as potential agents to inhibit lipid peroxidation and protect neuronal integrity (Liu et al., 2024; Zhang et al., 2023a). Their application mitigates ferroptotic pathways and offers neuroprotection, particularly in regions of high iron accumulation like the substantia nigra (Zhang et al., 2024a) and hippocampus (Lai et al., 2022).

The complex crosstalk between ferroptosis and neurodegeneration underscores the necessity of targeting this pathway for therapeutic development. Future research should focus on refining strategies to modulate ferroptosis while ensuring efficient blood–brain barrier (BBB) penetration of therapeutic agents. Novel delivery mechanisms, such as nanoparticle conjugates, represent a promising frontier in ferroptosis research.

### 2.4 Interplay between pathways: toward a unified proteostasis network

*Key takeaways:*

- *UPS, autophagy and ferroptosis are interconnected*
- *Treatment aimed at one mechanism may improve others*

The interplay between autophagy, ferroptosis, and the UPS highlights the dynamic and interconnected nature of proteostasis. For instance, when proteasomal activity declines, autophagic pathways may be upregulated to compensate for the reduced protein degradation capacity. Conversely, ferroptotic stress driven by lipid peroxidation can overwhelm both autophagy and the UPS, resulting in proteostatic collapse and neurodegeneration.

Ferritinophagy serves as a critical link between autophagy and ferroptosis (Biasiotto et al., 2016). By degrading ferritin and releasing iron, it can tip the balance toward ferroptotic cell death under conditions of oxidative stress. This underscores the intricate crosstalk between iron metabolism, autophagy, and proteostatic balance. This interconnectedness points to the need for multi-targeted therapeutic strategies that address the broader proteostasis network rather than isolated pathways. For example, treatments aimed at limiting ferritinophagy or mitigating lipid peroxidation through ferroptosis inhibitors (e.g., ferrostatin-1) may not only protect against ferroptosis but also preserve the functionality of autophagic and proteasomal pathways (Boag et al., 2022). By restoring balance among these mechanisms, it may be possible to mitigate neurotoxicity, enhance cellular resilience, and slow the progression of neurodegenerative diseases.

Understanding the roles and interplay of autophagy, ferroptosis, and the UPS in maintaining proteostasis provides critical insights into the pathogenesis of neurodegenerative diseases. These interconnected pathways are not only central to neuronal survival but also represent promising targets for therapeutic intervention. Targeting specific vulnerabilities within this network, such as the labile iron pool driving ferroptosis or oxidative damage impairing autophagy, could lead to more effective interventions for conditions like AD, PD, and ALS. Future research aimed at elucidating the nuances of these processes could pave the way for innovative strategies to restore proteostasis, reduce neuronal vulnerability, and improve outcomes for patients affected by these debilitating conditions.

## 3 NFE2L1, a master regulator of proteostasis

*Key takeaways:*

- *NFE2L1 is a transcription factor crucial for proteostasis*

NFE2L1, also known as nuclear factor erythroid 2-related factor 1 (NRF1), is a key transcription factor crucial for maintaining cellular protein homeostasis, or proteostasis. It is characterized by its CNC-bZIP (Cap ‘N’ Collar basic Leucine Zipper) domain, which allows it to bind to specific DNA elements and activate the expression of genes critical for proteasome function and biogenesis (Kim et al., 2016; Liu et al., 2023). The CNC-bZIP family includes NFE2L1 and related transcription factors like NFE2L2/Nrf2, sharing structural domains crucial for DNA binding and dimerization (Kim et al., 2016). NFE2L1 uniquely integrates signals from cellular stress pathways to fine-tune proteasome biogenesis, ensuring proper degradation of misfolded or damaged proteins to maintain proteostasis (Liu et al., 2023). Beyond its structural features, NFE2L1’s regulation includes sophisticated post-translational processing, such as glycosylation and proteolytic cleavage, which enable its dynamic response to cellular stress (Liu et al., 2023; Zhang et al., 2024b). These modifications facilitate NFE2L1’s transition from the endoplasmic reticulum (ER) to the nucleus, where it initiates transcriptional programs to restore cellular equilibrium under proteotoxic stress.

### 3.1 NFE2L1 in proteasomal regulation and recovery

*Key takeaways:*

- *NFE2L1 regulates the “bounce-back response” for proteasome recovery under its impairment*
- *NFE2L1 transcriptionally controls proteasome subunits*
- *NFE2L1 controls the expression of deubiquitinating enzymes*
- *ER stress is mitigated through NFE2L1’s involvement*

Upon proteasome impairment, NFE2L1 undergoes proteolytic processing, allowing its nuclear translocation and activation of proteasome-related genes via antioxidant response elements (AREs) (Biswas and Chan, 2010). This process, known as the “bounce-back response,” restores proteasome biogenesis and supports cellular recovery during proteotoxic stress (Liu et al., 2023). NFE2L1 controls all of the proteasome subunits. Studies have revealed that the proteasome subunit genes regulated by NFE2L1, including those encoding the 20S core and 19S regulatory complex, are essential for maintaining protein degradation capacity under stress (Chandran et al., 2023; Lemmer, 2023). Furthermore, NFE2L1 interacts with components of the ER-associated degradation (ERAD) pathway, such as Herpud1 and VCP/p97, which facilitate proteasome assembly and function during stress recovery (Ho and Chan, 2015; Sha and Goldberg, 2014).

Importantly, experimental findings suggest that NFE2L1’s role extends beyond proteasomal recovery to broader cytoprotective mechanisms. It transcriptionally controls genes encoding deubiquitinating enzymes (DUBs), which regulate ubiquitin chain dynamics, preventing the accumulation of ubiquitinated protein aggregates (Taniguchi et al., 2017; Han et al., 2021). Taniguchi et al. highlighted that in NFE2L1 knockout (Nrf1 NKO) mice, the absence of NFE2L1 led to reduced expression of DUB genes such as Usp9x, despite normal proteasome subunit activity (Taniguchi et al., 2017). This dysfunction resulted in the accumulation of ubiquitinated proteins in neurons, driving neurodegeneration. These findings underscore NFE2L1’s critical role in maintaining protein homeostasis and protecting neural tissue from proteotoxic stress.

Moreover, NFE2L1 has been shown to modulate the expression of key genes involved in mitigating ER stress-induced apoptosis. Li et al. demonstrated that Icariin, a natural flavonoid with neuroprotective properties, upregulates Synoviolin (Hrd1), an ER-resident E3 ubiquitin ligase, by increasing NFE2L1 binding to the distal promoter of the Synoviolin gene. This binding is essential for Icariin’s action, as mutations in these sites abolished Synoviolin promoter activation, and knockdown of NFE2L1 prevented its upregulation. More importantly, they found that Icariin protects neuronal cells from ER stress-induced apoptosis in a Synoviolin-dependent manner, suggesting a novel mechanism by which NFE2L1 contributes to neuronal survival under stress (Li et al., 2015).

The significance of NFE2L1-mediated proteostasis becomes particularly evident in the context of neurodegenerative diseases such as AD and PD, where impaired proteostasis is a hallmark of pathogenesis. Taniguchi et al. emphasized that while proteasome activity remained largely intact in NFE2L1-deficient cerebellar neurons, the suppression of DUB expression led to a compromised ability to manage ubiquitin-dependent proteostasis, further exacerbating neurodegeneration (Taniguchi et al., 2017). These findings, corroborated by various experimental models, suggest that therapies targeting NFE2L1 pathways may offer promising strategies for restoring neuronal integrity under proteotoxic and ER stress conditions.

**Figure 2:**
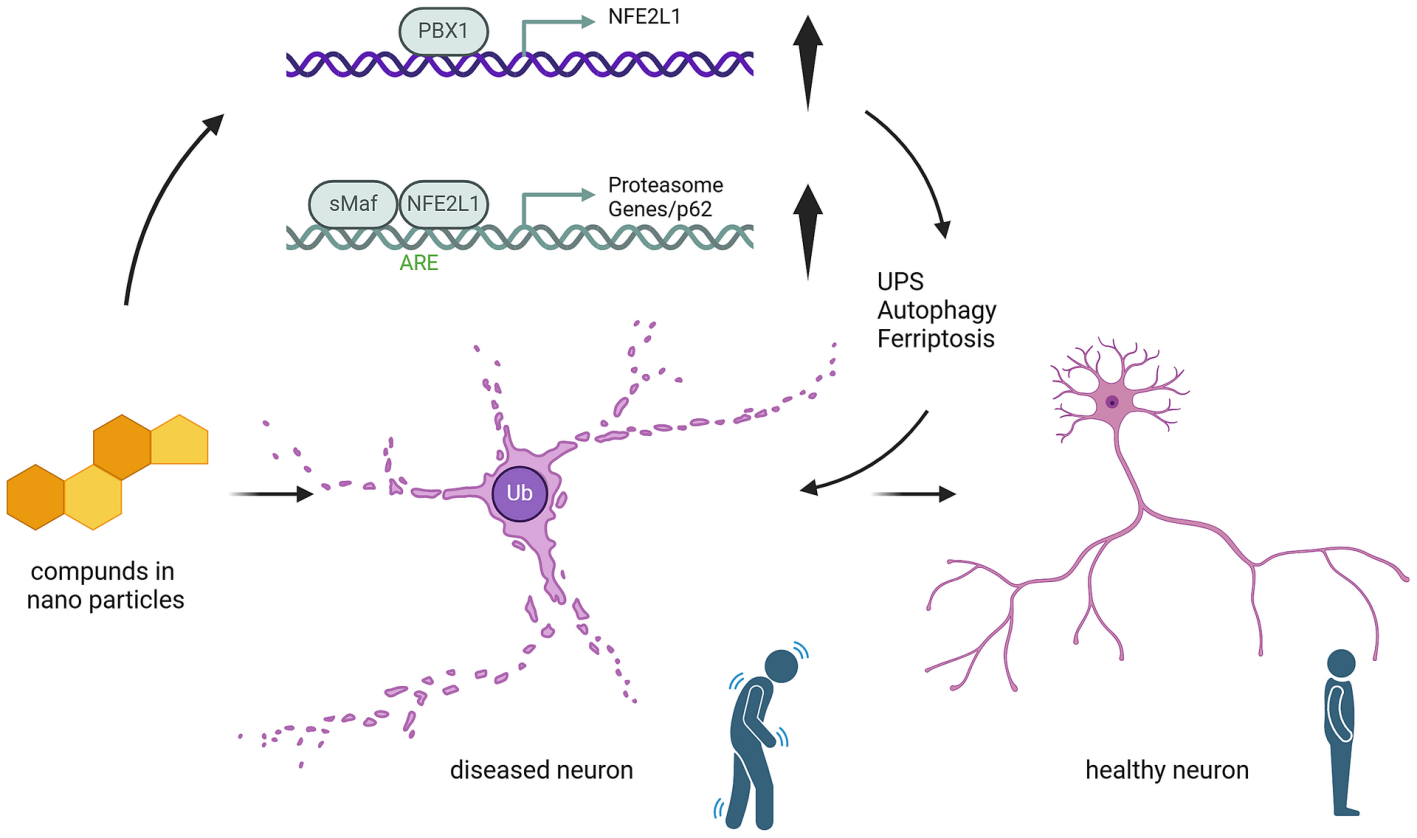
Mechanistic pathway of nanoparticle compounds in modulating NFE2L1-mediated neuroprotection. This schematic illustrates the effect of nanoparticle-based compounds on NFE2L1 and its downstream molecular pathways involved in neuronal health. Nanoparticle compounds (represented by compound molecules) potentially could activate the NFE2L1 pathway, leading to the upregulation of PBX1/NFE2L1 transcriptional activity. This activation enhances the Antioxidant Responsive Element (ARE), promoting the expression of antioxidant genes and protecting neurons from oxidative damage. Furthermore, upregulation of sMAF/NFE2L1 induces the activation of proteasomal degradation pathways (including p62-mediated autophagy), facilitating the clearance of damaged proteins and cellular debris. These events contribute to the regulation of ubiquitin-proteasome system (UPS) and autophagy, which are critical for maintaining neuronal homeostasis. The figure also shows the potential cellular impact: healthy neurons are maintained by these protective pathways, while diseased neurons, characterized by ubiquitinated proteins, are at risk of undergoing ferroptosis due to impaired oxidative stress responses and protein clearance. The upregulation of NFE2L1 serves as a potential therapeutic target to modulate these pathways and prevent neurodegeneration.

Experimental data from neuronal models confirm that NFE2L1 ensures the survival and functional integrity of neurons by mitigating proteotoxicity. By regulating both proteasomal activity and broader ubiquitin-related pathways, NFE2L1 emerges as a master regulator of cellular resilience against proteotoxic and ER stress, offering potential avenues for therapeutic intervention (Figure **2**).

### 3.2 NFE2L1 role in oxidative stress and ferroptosis

*Key takeaways:*

- *NFE2L1 governs activation of redox-related genes*
- *Absence of NFE2L1 sensitizes cells to ferroptosis*
- *NFE2L1 upregulates GPX4, decreasing lipid peroxidation*

NFE2L1 also plays a pivotal role in mitigating oxidative damage. It governs the transcriptional activation of redox-related genes, such as HMOX1 and glutathione synthesis enzymes, reinforcing cellular defenses against ROS (Ning et al., 2024; Liu et al., 2023). NFE2L1’s dual role in redox homeostasis and proteasome-mediated clearance provides a robust protective mechanism under oxidative stress. Notably, the transcriptional regulation of ARE-driven genes by NFE2L1 overlaps with NFE2L2, but NFE2L1 is uniquely activated in response to proteasome dysfunction, indicating complementary roles in cellular protection (Qiu et al., 2022; Ning et al., 2024). Recent studies have further illuminated NFE2L1’s multifaceted roles, particularly in ferroptosis (Ofoghi et al., 2024; Forcina et al., 2022). These findings underscore the importance of NFE2L1 not only in maintaining proteostasis under oxidative stress but also in mitigating ferroptotic damage through proteasome regulation and antioxidant mechanisms.

Ofoghi et al. (2024) reported that NFE2L1 activation is a pivotal adaptive response to ferroptosis, characterized by iron-dependent lipid peroxidation and cell death. Their findings emphasized that ferroptotic inducers, such as (1S,3R)-RSL3, not only impair proteasome activity but also result in the accumulation of ubiquitylated proteins, which triggers NFE2L1 activation via proteolytic cleavage by DDI2. This mechanism ensures a feedback loop that restores proteasomal activity by upregulating proteasome subunit genes. Additionally, NFE2L1 activation mitigates ferroptosis by maintaining proteostasis and alleviating oxidative damage.

Forcina et al. (2022) also characterized on this role, demonstrating that NFE2L1 activation is governed by post-translational modifications, particularly deglycosylation by N-glycanase 1 (NGLY1) and cleavage by DDI2. These steps are essential for its ability to promote the degradation of oxidatively damaged proteins and sustain levels of anti-ferroptotic factors like GPX4. The absence of NFE2L1 or disruptions in its activation pathways heightened cellular vulnerability to ferroptosis, particularly in conditions of increased lipid ROS and impaired proteasome function. These insights underscore the intricate regulatory network involving NFE2L1 in counteracting oxidative stress and ferroptosis.

**Figure 3:**
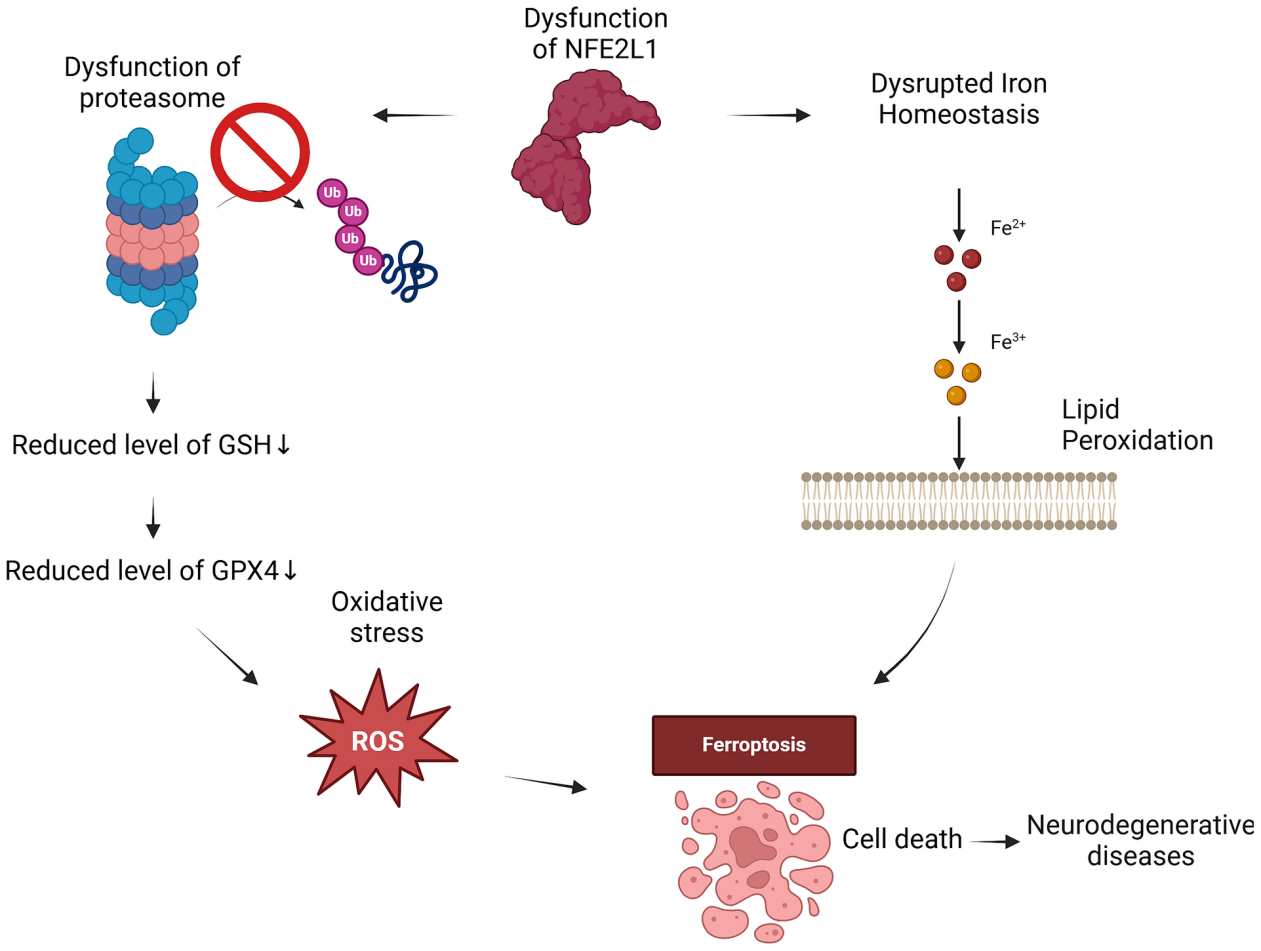
NFE2L1 dysfunction drives ferroptosis through iron dysregulation and oxidative stress. When NFE2L1 is dysfunctional, iron homeostasis is disrupted, leading to iron accumulation, which drives lipid peroxidation and ROS generation. The resulting oxidative damage weakens cellular membranes, while proteasome dysfunction leads to the accumulation of damaged proteins and oxidative stress. The loss of GPX4 activity and reduced GSH levels further impair antioxidant defenses, making neurons highly susceptible to ferroptosis. This cascade of events can contribute to neurodegeneration in conditions such as AD, PD and ALS.

Intriguingly, studies demonstrate that NFE2L1 activation during oxidative stress not only enhances proteasome-mediated protein clearance but also stabilizes proteins essential for combating ferroptosis, an iron-dependent form of cell death driven by lipid peroxidation (Kotschi et al., 2022; Zhang et al., 2024b). Under ferroptotic conditions, NFE2L1 facilitates the transcriptional activation of proteasome subunits critical for degrading misfolded or oxidatively damaged proteins, a process essential for preserving cellular homeostasis (Kotschi et al., 2022). It also upregulates GPX4, a key regulator of ferroptosis, thereby mitigating lipid ROS accumulation (Forcina et al., 2022). This dual functionality ensures cellular survival during oxidative stress by balancing redox homeostasis and proteostasis. Loss of NFE2L1 exacerbates vulnerability to ferroptosis, as seen in models exhibiting severe proteasome dysfunction and increased lipid peroxidation, underscoring its adaptive role during oxidative and metabolic stress (Figure 3).

## 4 NFE2L1 and autophagy: ensuring cellular clean-up and protein recycling

*Key takeaways:*

- *NFE2L1 is required for autophagy and aggrephagy*
- *NFE2L1 is involved in the formation of p62-positive puncta*
- *NFE2L1 activates autophagy-related genes*
- *Overexpression of NFE2L1 improves autophagy*

While UPS is a major protein disposal system, it mainly deals with relatively small proteins, able to enter into the proteasome (Pohl and Dikic, 2019). For disposal of large structures, cells utilize another pathway that enables the target to be engulfed in the vacuole and “consumed.” Similarly to UPS, autophagy defects have been found to be widespread in neurodegenerative disorders and understanding the regulation of this pathway is essential for developing prevention strategies and treatments. In this section, we will describe the place of NFE2L1 in ensuring a healthy autophagy process.

A study by Hatanaka et al. demonstrated that NFE2L1 knockdown in HCT116 cells altered the clearance of ubiquitinated proteins following proteasome inhibition with MG132 (Hatanaka et al., 2023). Treatment with the autophagy inhibitor bafilomycin A1 (BafA) led to increased ubiquitinated protein accumulation, confirming that autophagy plays a role in degrading these proteins. However, in NFE2L1-deficient cells, BafA treatment did not further increase protein accumulation, suggesting that NFE2L1 is specifically required for aggrephagy, a selective form of autophagy that clears protein aggregates. These results indicate that while autophagy can compensate for proteasome dysfunction in normal cells, NFE2L1 is necessary for effective aggrephagy under proteotoxic stress.

Further mechanistic insights revealed that NFE2L1 is involved in the formation of p62-positive punctum. Treatment with the proteasome inhibitor MG132 increased the formation of p62-positive puncta in wild-type cells, whereas NFE2L1 knockdown abolished this increase. Interestingly, while p62 protein was still present in NFE2L1-deficient cells, it failed to form puncta. In healthy cells, p62 phosphorylation at Ser403 is required for puncta formation; however, NFE2L1 knockdown reduced phosphorylation at this site. Additionally, GABARAPL1 protein expression was decreased following NFE2L1 knockdown, further suggesting its role in autophagy regulation. Interestingly, another study revealed both similar and differing findings. This study demonstrated that NFE2L1 activates p62 in multiple cell lines, including SH-SY5Y, HAP1, M17, and HEK293A, whereas NFE2L2 failed to do so (Sha et al., 2018). However, NFE2L1-deficient cells exhibited deficiencies not only in the UPS but also in the autophagy-lysosome pathway (ALP). Proteasome inhibition upregulated several ALP-related genes, including GABARAPL1, VPS37A, ATG4A, and CTSD, but this response was significantly attenuated in NFE2L1-deficient cells (Ward et al., 2024). These findings were confirmed across multiple cell lines, including human breast cancer MDA-MB-231 cells, human neuroblastoma SH-SY5Y cells, and murine hippocampal neurons HT22, demonstrating that this effect is also present in neuronal cells. At the protein level, reduced expression of GABARAPL1, VPS37A, and Cathepsin D was observed in NFE2L1-deficient cells. Furthermore, chromatin immunoprecipitation (ChIP) experiments confirmed that NFE2L1 binds to the ARE motif in the GABARAPL1 promoter, recruiting transcriptional machinery in response to proteasome inhibition (Ward et al., 2024). The promoters of GABARAPL1, CTSD, and VPS37A were all activated following NFE2L1 overexpression, supporting its role in transcriptional regulation of autophagy genes.

Despite gene upregulation, functional assays were necessary to confirm NFE2L1’s impact on autophagy. Transmission electron microscopy (TEM) revealed increased autophagosomal structures and autophagic vacuoles in NFE2L1-deficient cells, indicating an impairment in autophagic flux (Ward et al., 2024). This effect was further validated in HT22 neurons, where NFE2L1 knockout led to an attenuation of autophagic flux. Additionally, NFE2L1 was implicated in aggrephagy, the selective clearance of protein aggregates. In HT22 wild-type cells, proteasome inhibition caused aggresome formation, but after returning to fresh medium, the aggregates were successfully cleared. In contrast, NFE2L1-deficient HT22 cells exhibited impaired aggresome clearance, reinforcing its role in autophagy-mediated degradation. Notably, overexpression of GABARAPL1 or p62/SQSTM1 alone failed to rescue aggresome clearance in NFE2L1-deficient cells (Ward et al., 2024), suggesting that NFE2L1 regulates autophagy through multiple mechanisms.

Finally, NFE2L1 overexpression alone, even in the absence of proteasomal stress, was sufficient to activate autophagy. In these cells, transcript and protein levels of GABARAPL1, CTSD, VPS37A, and ATG4A increased, along with a rise in autolysosome formation. Moreover, aggresome clearance efficiency significantly improved, demonstrating that NFE2L1 actively promotes autophagy under both normal and stress conditions (Ward et al., 2024; Sha et al., 2018).

Together, these findings establish NFE2L1 as a key regulator of autophagy, influencing both the transcription of autophagy-related genes and functional autophagic flux.

## 5 Mitochondrial protein homeostasis and mitophagy: the essential role of NFE2L1 in neurons and other systems

*Key takeaways*:

- *Cells lacking NFE2L1 show downregulation of several mitochondria-related genes*
- *NGLY1 regulates mitochondrial homeostasis via its activation of NFE2L1*
- *NFE2L1 is highly expressed in brown adipose tissue and its expression correlates with UCP1*
- *Brown adipose tissue lacking NFE2L1 showed significant mitochondria impairment*

Although the brain accounts for only 2% of body weight, it consumes 20% of the body’s oxygen (Herculano-Houzel, 2011; Trigo et al., 2022). Neurons require a substantial amount of energy to maintain resting potential, generate action potentials, and conduct neurotransmission, among other energy-intensive processes. As a result, neurons contain numerous mitochondria, and proper mitochondrial function is critical to their overall health and function. One key aspect of mitochondrial homeostasis is the disposal of damaged mitochondria, primarily through mitophagy, an autophagy process specific to mitochondria. Dysregulation of mitophagy is a fairly established event during the course of neurodegenerative diseases (Jetto et al., 2022), to the point that one of the major proteins involved in mitophagy was named in connection to Parkinson’s disease, Parkin.

A significant part of mitochondrial homeostasis involves NFE2L1, a key transcription factor regulating mitochondrial function and mitophagy. Cells deficient in NFE2L1 show downregulation of several mitochondrial homeostasis-related genes, including nuclear respiratory factor 1 (also abbreviated to NRF1, despite being a different gene), and mitochondrial transcription factor A (TFAM) (Zhang et al., 2020). However, to fully understand NFE2L1’s role in mitochondrial integrity, it is important to examine the enzyme NGLY1, which is essential for the activation of NFE2L1.

NGLY1 is a deglycosylation enzyme responsible for removing N-glycans from misfolded glycoproteins, thus ensuring proper protein quality control (Suzuki et al., 2016). Mutations in the NGLY1 gene cause congenital disorders, leading to neurological and liver dysfunctions, including movement disorders and developmental delays (Enns et al., 2014). This disease is typically categorized as a disorder of the ER-associated degradation (ERAD) pathway. Further studies have revealed that variants of NGLY1 are linked to intellectual disabilities, peripheral polyneuropathy, and mitochondrial dysfunction (Panneman et al., 2020). Notably, recent findings show that NGLY1 plays a critical role in maintaining mitochondrial homeostasis, including the clearance of dysfunctional mitochondria (Kong et al., 2018). Neurons derived from iPSCs from patients with NGLY1 mutations display protein aggregation, synaptic dysfunction, and mitochondrial abnormalities (Manole et al., 2023).

An important study by Yang et al. found that NGLY1 is essential for mitochondrial homeostasis through its regulation of NFE2L1 (Yang et al., 2018). For NFE2L1 to translocate from the cytosol to the nucleus and function as a transcription factor, it requires deglycosylation by NGLY1. In the absence of NGLY1, NFE2L1 becomes trapped in the cytosol, unable to perform its critical functions. As a result, NGLY1-deficient cells display a similar phenotype to what is observed in NFE2L1-deficient cells, including a lack of proteasome activation and mitochondrial dysfunction. Yang et al. also created a model in which NFE2L1 was activated independently of NGLY1. These cells, expressing NFE2L1 without its ER-anchor, showed restored proteasome activity and improved mitochondrial homeostasis, demonstrating that activation of NFE2L1 could rescue the phenotypic effects of NGLY1 deficiency. Interestingly, similar effects were observed when NFE2L2 expression was enhanced using known pharmacological activators.

Studies involving brown adipose tissue (BAT) have provided further insights into the role of NFE2L1 in mitochondrial integrity. BAT is responsible for thermogenesis and is characterized by the presence of uncoupling protein 1 (UCP1), which allows energy conversion into heat rather than ATP (Oelkrug et al., 2015). It has been documented that NFE2L1 is highly expressed in human primary differentiated brown adipocytes, and its expression level correlates strongly with UCP1 expression (Bartelt et al., 2018; Ren et al., 2021). Bartelt et al. (2018) highlighted the role of NFE2L1 in maintaining mitochondrial integrity in BAT. They demonstrated that NFE2L1 is highly expressed in BAT, particularly during cold exposure, and is crucial for mitochondrial homeostasis and thermogenic function. Nfe2l1ΔBAT mice, with NFE2L1 disrupted in UCP1-expressing cells, exhibited cold intolerance and reduced survival rates under sustained cold conditions. These mice also displayed significant mitochondrial dysfunction, characterized by ultrastructural abnormalities such as swollen mitochondria, disorganized cristae, and lipid accumulation. Single-nucleus RNA sequencing (snRNA-seq) further revealed altered cellular heterogeneity in BAT, with reduced thermogenic cell populations and increased inflammation-associated subpopulations (Shen et al., 2023).

Shen et al. also observed the downregulation of proteasome and lipolytic genes, including ATGL, HSL, and PLIN1, which led to lipid accumulation and reduced BAT functionality. Functional assays showed that mitochondria from Nfe2l1ΔBAT mice exhibited reduced respiratory capacity, including diminished UCP1-dependent respiration and oxidative phosphorylation activity. These findings underscore the central role of NFE2L1 in mitochondrial homeostasis and thermogenesis.

Moreover, the absence of NFE2L1 in BAT led to hyperubiquitination, which aligns with NFE2L1’s role in the UPS (Bartelt et al., 2018; Liu et al., 2023). Notably, 34% of the hyperubiquitinated proteins were identified in the mitochondrial proteome (Mitocarta) as involved in mitochondrial function, including UCP1 and elements of the respiratory chain. Similar mitochondrial dysfunction was observed when NFE2L1 was knocked out in hepatic cells (Hirotsu et al., 2012) and insulinoma cells (Fu et al., 2018), where NFE2L1 deficiency resulted in dysregulation of the mitochondrial respiratory chain and increased mitochondrial membrane potential.

In conclusion, inhibiting NFE2L1, either by genetic knockout or by trapping it in the cytosol, results in severe mitochondrial dysfunction. This dysfunction is characterized by mitochondrial structural abnormalities and decreased respiratory capabilities. In the case of NGLY1 mutations, this dysfunction leads to neurological consequences. However, the full scope of mitochondrial damage caused by NFE2L1 dysfunction in neurons remains unclear and warrants further investigation.

## 6 The link between NFE2L1 and ferroptosis in neurodegenerative diseases

*Key takeaways:*

- *Ferroptosis is characterized by the failure of antioxidant defence system*
- *NFE2L1 transcriptionally governs multiple redox related genes*
- *NFE2L1 activation may be a way to mitigate ferroptosis*

Controlled cell death is crucial for development and the maintenance of homeostasis. Extensive cell death may lead to pathologies such as neurodegeneration, while on the other hand disruptions in this process can promote tumorigenesis. Apoptosis is the most studied type of cell death; however, other mechanisms such as pyroptosis, necroptosis, and ferroptosis also contribute to cell death (Murray and Dixon, 2024; Yuan and Ofengeim, 2024).

Ferroptosis is distinct from other forms of cell death, owing to its reliance on iron and the accumulation of lipid peroxides (Dixon et al., 2012). It is characterized by the failure of antioxidant defense systems, leading to an overwhelming burden of ROS and subsequent lipid peroxidation (Dixon et al., 2012; Yang et al., 2014). Ferroptosis has been implicated in various neurodegenerative diseases, where oxidative stress plays a pivotal role in neuronal degeneration (Reichert et al., 2020). Elevated iron levels in the brain enhance ROS production, leading to extensive lipid peroxidation and subsequent neuronal cell death (Dixon et al., 2012).

Iron-dependent enzymes such as lipoxygenases play a critical role in ferroptosis by catalyzing lipid peroxidation. The accumulation of oxidized lipids destabilizes cell membranes and triggers cell death (Dixon et al., 2012). Ferroptosis is tightly regulated by antioxidant systems, notably GPX4, which reduces lipid hydroperoxides. In oxidative stress scenarios, like neuroinflammation or mitochondrial dysfunction, these defenses fail, increasing neuronal susceptibility to ferroptosis (Kotschi et al., 2022; Forcina et al., 2022).

Researches suggest that this process contributes significantly to the pathophysiology of neurodegenerative diseases, as cells in regions of the brain most affected by these diseases exhibit increased levels of iron and lipid peroxides, pushing the system into ferroptotic pathways (Dixon et al., 2012; Buijs et al., 2017).

While many researches focus on the mechanisms driving ferroptosis in neurodegeneration, emerging evidence suggests a potential role for NFE2L1 in mitigating these effects (Murray and Dixon, 2024, Yuan and Ofengeim, 2024). By enhancing antioxidant defenses and regulating iron and lipid metabolism, NFE2L1 may influence susceptibility to ferroptosis. Neurodegenerative diseases such as AD, PD and HD have been associated with disrupted iron homeostasis, increased oxidative stress, and lipid peroxidation, all hallmarks of ferroptosis (Ji et al., 2022; Wang et al., 2024) (Figure 3).

These findings suggest that NFE2L1 may act as a key modulator in ferroptosis in neurodegenerative diseases, highlighting its potential as a therapeutic target. As outlined in Table **1**, NFE2L1 regulates multiple key pathways involved in ferroptosis, including iron metabolism, antioxidant defense, lipid metabolism, and neuroinflammation. Dysregulation of these processes due to NFE2L1 dysfunction has been implicated in several neurodegenerative diseases such as AD, PD, and HD.

**Table 1:** NFE2L1’s impact on ferroptosis regulation in neurodegenerative diseases.

Pathway/Function	Effect of NFE2L1 dysfunction	Neurodegenerative diseases affected
Iron metabolism regulation	Disruption of iron homeostasis, promoting ROS production	Alzheimer’s (AD), Parkinson’s (PD), Huntington’s (HD)
Antioxidant defenses (GPX4 regulation)	Reduced antioxidant activity, leading to lipid peroxidation	PD, AD
Lipid metabolism (PUFA regulation)	Altered lipid profiles, increasing lipid peroxidation	AD, HD
Neuroinflammation modulation	Chronic activation of microglia and astrocytes, promoting oxidative stress and ferroptosis	AD, PD

The ability to modulate NFE2L1 activity opens up new avenues for therapeutic intervention, either through direct NFE2L1 activation or through the development of ferroptosis inhibitors that reduce lipid peroxidation. Targeting these pathways could complement existing treatments and offer a powerful strategy to slow down or even reverse the progression of neurodegenerative diseases, potentially improving quality of life for affected individuals.

### 6.1 Potential interactions between NFE2L1 function and ferroptosis regulation

*Key takeaways:*

- *NFE2L1 influences the activity of GPX4, which protects cells from lipid peroxidation*
- *NFE2L1 regulates genes tied to iron metabolism*
- *NFE2L1 regulates enzymes in the biosynthesis of PUFAs and phospholipids*

During oxidative stress, NFE2L1 enhances antioxidant defenses, reducing lipid peroxidation and the risk of ferroptosis. NFE2L1 has been shown to influence the activity of GPX4, the major enzyme that protects cells from oxidative stress and lipid peroxidation (Forcina et al., 2022; Kotschi et al., 2022), moreover, research highlights NFE2L1’s influence on ferroptosis by regulating genes that are tied to iron metabolism (Tang et al., 2018). Beyond its role in iron metabolism and antioxidative protection, NFE2L1 may also influence lipid metabolism pathways that affect ferroptosis (Widenmaier et al., 2017; Tsujita et al., 2014). It regulates enzymes involved in the biosynthesis of polyunsaturated fatty acids (PUFAs) and phospholipids, the major substrates for lipid peroxidation (Zhao et al., 2024; Muley et al., 2021). Thus, a balanced lipid profile, modulated by NFE2L1, may prevent excessive lipid peroxidation, further reducing ferroptosis susceptibility.

While NFE2L1’s involvement in mitochondrial function is recognized, its role extends beyond just redox balance. NFE2L1 helps modulate the expression of various genes that regulate mitochondrial energy production, and antioxidant defense mechanisms, all of which are crucial for cell survival under stress conditions. Mitochondria, often referred to as the powerhouses of the cell, are not only responsible for energy production but also for regulating the generation of ROS. Elevated ROS production, especially under pathological conditions, can overwhelm the cell’s antioxidant defenses, exacerbating lipid peroxidation and triggering ferroptosis (Forcina et al., 2022, Kotschi et al., 2022). Therefore, mitochondrial dysfunction becomes a key factor in ferroptosis, as it contributes directly to the accumulation of damaging ROS.

NFE2L1 plays a crucial role in activating cellular responses to oxidative stress through the upregulation of antioxidant genes and mitochondrial repair pathways. By regulating the transcription of genes involved in mitochondrial maintenance, such as those responsible for mitophagy and mitochondrial biogenesis, NFE2L1 helps protect neurons from the harmful effects of oxidative damage, which is a central mechanism in neurodegenerative disorders. Moreover, in AD and PD, mitochondrial dysfunction leads to a vicious cycle of oxidative stress, impaired energy metabolism, and neuronal death. NFE2L1, by supporting mitochondrial biogenesis and stabilizing mitochondrial function, may provide a critical defence against these pathological changes. Its regulation of mitochondrial dynamics and energy production supports the survival of vulnerable neuronal populations, particularly in regions affected by these neurodegenerative diseases.

### 6.2 Analysis of possible NFE2L1 dysfunction and increased ferroptosis susceptibility in neurons

*Key takeaways:*

- *Dysfunction of NFE2L1 leads to an impairment of antioxidant defense*
- *Impaired antioxidant defence makes neurons more prone to oxidative damage*

Neurons are cells that are rich in phospholipids containing PUFA and iron, likely sensitizing them to ferroptosis. There is no direct connection between NFE2L1 and ferroptosis in neurons, but we can assume some links from other tissues. Dysfunction of NFE2L1 is implicated in several neurodegenerative diseases due to its inability to properly regulate oxidative stress responses and iron metabolism. In neurons, this dysfunction may disrupt the balance of iron, ROS and lipid homeostasis, fostering conditions conducive to ferroptosis (Jacquemyn et al., 2024; Liu et al., 2023).

NFE2L1 can regulate genes responsible for iron metabolism (Jacquemyn et al., 2024). Excess iron triggers the Fenton reaction, a process that generates ROS and accelerates lipid peroxidation, initiating ferroptosis (Gaschler and Stockwell, 2017). Additionally, when NFE2L1 function is compromised, antioxidant defense mechanisms become ineffective. In such conditions, GPX4 activity may be reduced, allowing the accumulation of lipid peroxides and making neurons more prone to oxidative damage as seen in cancer cell models (Yang et al., 2014). Moreover, biosynthesis of PUFAs and phospholipids may be controlled by NFE2L1 (Widenmaier et al., 2017; Zhao et al., 2024; Tsujita et al., 2014), the major substrates for lipid peroxidation may be altered in neurons with dysfunction of this crucial transcriptional factor. Without new lipids, peroxidation of existing phospholipids may be excessive, pushing neurons into ferroptosis pathway. This deficiency may be particularly damaging in the context of neurodegeneration, where neurons are already under oxidative stress due to mitochondrial dysfunction, inflammation, and age-related changes linking this with NFE2L1 dysfunctions may reveal new therapeutic potential in AD or PD.

NFE2L1 may play a critical role in regulating the inflammatory response within neurons and glial cells as it is connected with anti-inflammatory effect in adipocytes (Muley et al., 2021). The above-mentioned dysfunctions may lead to the disruption of neuroinflammatory homeostasis, resulting in chronic activation of microglia and astrocytes (Zhang et al., 2023b). This activation exacerbates oxidative stress and inflammatory signaling, creating an environment conducive to ferroptosis. Under normal conditions, microglial activation is a protective response to injury or disease, but chronic activation seen in neurodegenerative diseases such as AD and PD leads to excessive production of pro-inflammatory cytokines like TNF-*α* and IL-1β (Zhang et al., 2023b). These cytokines amplify oxidative stress in neurons, impairing cellular repair mechanisms and enhancing lipid peroxidation, which pushes the affected neurons toward ferroptotic cell death. This inflammation-driven ferroptosis can contribute significantly to disease progression. Neurons’ impaired ability to counteract this stress, due to possible NFE2L1 dysfunction, may increase their vulnerability to ferroptosis and cell death.

NFE2L1 regulates neuroinflammation, iron homeostasis, and oxidative stress, key factors in neurodegenerative diseases. Its dysfunction may disrupt iron metabolism and antioxidant defenses, increasing neuronal susceptibility to ferroptosis. By impairing ROS detoxification and lipid homeostasis, reduced NFE2L1 activity can contribute to oxidative damage and cell death. Targeting NFE2L1 through pharmacological activation or gene therapy could help restore redox balance, limit iron-induced toxicity, and reduce lipid peroxidation. Understanding the extent of NFE2L1’s neuroprotective role, also could provide new therapeutic strategies for conditions such as AD and PD.

## 7 Pathological implications of NFE2L1 dysfunction in neurodegenerative diseases

In previous sections, we described how NFE2L1 governs multiple pathways cells use to dispose of misfolded and unnecessary proteins, including regulating proteasomal function, autophagy, ferroptosis, and mitochondrial homeostasis. Given that neurodegenerative diseases are characterized by the accumulation of misfolded, heavily ubiquitinated proteins, it is hypothesized that dysfunction of NFE2L1 may contribute to the pathogenesis and progression of these diseases. Indeed, *in vitro* studies on neuronal and neuron-like cells show that when NFE2L1 is compromised, ubiquitinated protein accumulation, functional impairment, and cell death occur. However, whether these findings are consistent across whole organisms and in humans remains unclear. This section will present evidence supporting a correlation, and possibly a causal link, between NFE2L1 dysfunction and neurodegenerative diseases, as observed in knockout mouse models and postmortem brain studies.

### 7.1 NFE2L1 knockout models and neurodegeneration

*Key takeaways:*

- *NFE2L1 KO in neurons via Cre-Lox models leads to neurodegeneration*
- *NFE2L1 KO in Nestin-Cre model causes death by three weeks of age*
- *NFE2L1 KO in forebrain-specific model causes significant brain atrophy in that region*

*In vivo* evidence for the involvement of NFE2L1 in neurodegenerative processes comes from conditional knockout (KO) models, which allow for the selective deletion of NFE2L1 in the nervous system. One significant study by Kobayashi et al. (2011) utilized a Nestin-Cre system to delete NFE2L1 across the nervous system. The mice exhibited severe neurodegeneration and early death by three weeks of age. Histological analyses revealed significant damage in the hippocampus, particularly in the CA3 region, and the accumulation of ubiquitinated proteins, indicative of impaired proteasomal function. Oxidative stress markers such as heme oxygenase-1 (HO-1) and lipid peroxidation products like 4-hydroxy-2-nonenal (4-HNE) were elevated, further supporting the hypothesis that NFE2L1 dysfunction exacerbates neurodegeneration (Kobayashi et al., 2011).

In another study, Lee et al. (2011) used a forebrain-specific NFE2L1 KO model, demonstrating that NFE2L1 deficiency resulted in behavioral deficits such as hindlimb clasping by 3–4 months of age. By 6 months, significant atrophy was observed in the cortex and hippocampus, regions that are particularly vulnerable to impaired neuronal function. Interestingly, this model did not exhibit elevated oxidative stress, suggesting that NFE2L1’s role in neurodegeneration may extend beyond its antioxidant function (Lee et al., 2011).

As summarized in Table 2, these *in vivo* models across different species and genetic alterations provide a clearer picture of the functional impact of NFE2L1 dysfunction in neurodegenerative diseases. The table highlights various findings from knockout studies in mice and other organisms, as well as postmortem analyses in humans, providing a broader view of how NFE2L1 alterations can contribute to neurodegeneration.

**Table 2:** *In vivo* evidence of NFE2L1 dysfunction in neurodegeneration.

Species	Gene/Ortholog	Study context	Genetic alterations	Phenotypic outcomes	Reference
Human	Human NFE2L1	Postmortem PD patients	Reduced expression NFE2L1 in SN	Functional effects unknown	Villaescusa et al. (2016)
Human	Human NFE2L1	Postmortem AD brains	Reduced NFE2L1 expression in hippocampus	Functional effects unknown	Acquaah-Mensah and Taylor (2016)
Mice	Mice NFE2L1	Nervous system	Nestin-Cre NFE2L1 KO	Severe neurodegeneration, accumulation of ubiquitinated proteins, early death	Kobayashi et al. (2011)
Mice	Mice NFE2L1	Forebrain-specific	Camk2Cre NFE2L1 KO	Behavioral deficits, cortical and hippocampal atrophy	Lee et al. (2011)
*D. melanogaster*	CncC (Ortholog of NFE2L1–3)	Whole organism	CncC Deletion/RNAi	Dendritic pruning defects	Chew et al. (2021)
*D. melanogaster*	CncC (Ortholog of NFE2L1–3)	Whole organism	Overexpression of CncC in PINK/Parkin knockdown	Rescue of PINK/Parkin induced neuromuscular degenerative phenotypes, elevation of mitophagy rates, induction of proteostasis	Gumeni et al. (2021)
*C. elegans*	SKN-1 (Ortholog of NFE2L1–3)	Whole organism	skn-1(zu67) and skn-1(zu129) loss-of-function	Shortened lifespan, behavioral deficits	Turner et al. (2024), An and Blackwell (2003); Turner and Curran (2025); Wilson et al. (2017)

### 7.2 NFE2L1 dysfunction in Parkinson’s disease

*Key takeaways:*

- *NFE2L1 expression is reduced in substantia nigra of PD patients*

Parkinson’s disease, a neurodegenerative disorder characterized by the loss of dopaminergic neurons in the substantia nigra (SN), has been linked to deficits in NFE2L1 expression. Villaescusa et al. (2016) reported a significant reduction in NFE2L1 levels in the SN of postmortem PD brains, while other midbrain regions showed normal expression. This selective reduction suggests that NFE2L1 dysfunction in the SN may contribute to PD pathogenesis by exacerbating the accumulation of misfolded proteins such as *α*-synuclein, a hallmark of PD (Villaescusa et al., 2016). Additionally, studies by Lee et al. (2011) and Chan et al. (1998) emphasize the role of NFE2L1 in regulating oxidative stress and protein turnover in neurons, both of which are critical in PD development.

### 7.3 NFE2L1 dysfunction in Alzheimer’s disease

*Key takeaways:*

- *NFE2L1 expression in hippocampal CA1 in AD patients is reduced*
- *NFE2L1 is upregulated in surviving excitatory neurons of tauopathies*

In Alzheimer’s disease, a progressive neurodegenerative disorder marked by cognitive decline and amyloid-beta plaques, NFE2L1 expression has also been implicated. Acquaah-Mensah and Taylor (2016) reported reduced NFE2L1 expression in pyramidal neurons of the hippocampal CA1 region in postmortem AD brains, suggesting its role in AD pathophysiology. Further studies by Rexach et al. (2024) using single-nucleus RNA sequencing found that NFE2L1 expression was upregulated in surviving excitatory neurons across AD and other tauopathies, such as frontotemporal dementia (FTD) and progressive supranuclear palsy (PSP). This suggests that NFE2L1 might contribute to neuronal resilience in the face of tau-induced damage. However, the study also revealed that certain projection neurons in PSP were selectively depleted and showed reduced NFE2L1 activity, indicating a vulnerability specific to this disease (Rexach et al., 2024).

## 8 Therapeutic potential of targeting NFE2L1 for neurodegenerative diseases

NFE2L1 has emerged as a promising therapeutic target for neurodegenerative diseases due to its crucial role in regulating oxidative stress, protein turnover, and cellular resilience. Restoring NFE2L1 function could enhance proteasomal degradation of misfolded proteins, reduce oxidative stress, and protect against neuronal death. Postmortem studies of the SN in patients with PD revealed significantly reduced levels of NFE2L1 compared to controls (Łuczyńska et al., 2024; Villaescusa et al., 2016). Notably, this reduction was localized to the SN, while other brain regions remained unaffected. Another study leveraging the Allen Brain Atlas and ISH gene expression analysis on hippocampal tissue from mice identified NFE2L1 as a key regulator of genes involved in the pathogenesis of AD (Łuczyńska et al., 2024; Acquaah-Mensah and Taylor, 2016). The findings strongly suggest that reduced levels of NFE2L1 may play a causative role in the development of AD. Additionally, Wang et al. reported a heterozygous mutation (c.1855C > T) in the NFE2L1 gene that generates a truncated isoform due to a premature stop codon (Łuczyńska et al., 2024; Wang et al., 2022a). The patient carrying this mutation presented with global developmental delay, failure to thrive, and dysmorphic features by 17 months of age, underscoring the critical role of NFE2L1 in neurological development and function. These findings collectively highlight the pivotal role of NFE2L1 in maintaining neural homeostasis and its potential as a therapeutic target in neurodegenerative diseases.

### 8.1 NFE2L1 deficiency and neurodegeneration

*Key takeaways:*

- *NFE2L1 KO results in neurodegeneration*
- *Activation of NFE2L1 homolog cncC in D. melanogaster PD model protects from neurodegeneration*

Experimental models further highlight NFE2L1’s role in neuroprotection. In two studies, the neuronal specific knockout of NFE2L1 resulted in the accumulation of ubiquitinated proteins due to impaired protein turnover, leading to neurodegenerative symptoms such as motor ataxia and hindlimb clasping (Kobayashi et al., 2011; Lee et al., 2011). Therefore, identifying and specifically removing these ubiquitinated proteins caused by NFE2L1 deficiency is critical for preventing neuronal disorders caused by the loss of NFE2L1. Mutations in α-synuclein, particularly A53T, are linked to early-onset autosomal dominant PD. Transgenic flies expressing mutant α-synuclein (A53T) exhibit neurodegeneration, including movement impairments and dopaminergic neuron loss. Activation of the NFE2L1 homolog cncC in dopaminergic neurons protects against these neurodegenerative effects, suggesting α-synuclein may be a target of NFE2L1-mediated ubiquitination. Further studies are needed to confirm this mechanism (Wang et al., 2015).

### 8.2 Therapeutic strategies to enhance NFE2L1 activity

*Key takeaways:*

- *NFE2L1 activation may be a strategy to mitigate or prevent neurodegenerative disorders*
- *Several compounds have been shown to enhance NFE2L1 activity*
- *NFE2L1 activity may be enhanced by increasing NFE2L1 cleavage from ER*
- *It may be possible to enhance NFE2L1 activity via aspartyl protease DNA-damage inducible 1 homolog 2*

It is known that the complete loss of NFE2L1 is embryonically lethal (Chan et al., 1998), suggesting that heterozygous mutations or SNPs in one allele, or reduced expression or activity of NFE2L1, may drive specific neurological diseases. To address these issues, efforts to increase the activity or expression of NFE2L1 are essential. Although examples of NFE2L1 activation in neuronal cells are scarce, it is known that its expression is regulated by factors such as PBX1 (Villaescusa et al., 2016), though the detailed regulatory mechanisms in NFE2L1 gene regulation at the promoter and enhancer levels remain largely unexplored. Subsequent studies could involve administering small-molecule compounds to mice with neuron-specific NFE2L1 knockdown to investigate whether such interventions alleviate neurological diseases (Łuczyńska et al., 2024). Compounds such as vitamin E (Acquaah-Mensah and Taylor, 2016) have been reported to enhance NFE2L1 activity, making them potential candidates for testing. Another approach to activating NFE2L1 involves enhancing its cleavage from the ER. Research has shown that NFE2L1 is cleaved at a site N-terminal to Leu-104, resulting in a fragment that is no longer tethered to the ER membrane. This cleavage occurs in the presence of a proteasome inhibitor, suggesting that NFE2L1 is processed by a specific protease to execute its role in activating target genes in the nucleus (Radhakrishnan et al., 2014).

Excitingly, a recent study identified aspartyl protease DNA-damage inducible 1 homolog 2 as a protease responsible for cleaving NFE2L1 under conditions of RSL3-induced ferroptosis. This cleavage was shown to enhance NFE2L1’s transcriptional activity (Ofoghi et al., 2024). While further validation is required, this mechanism holds potential for clinical applications. Specifically, ferroptosis-inducing agents like RSL3 could be explored as therapeutic tools to activate NFE2L1 in patients with neuronal diseases.

Despite the promising therapeutic implications of targeting NFE2L1, challenges remain in translating these findings into clinical treatments. Much of the current research is in its infancy, with the need for more comprehensive *in vivo* and clinical studies to assess the efficacy and safety of NFE2L1-based therapies. Moreover, the exact regulatory mechanisms that control NFE2L1 expression and activity in the nervous system are still poorly understood, and more research is required to uncover these pathways.

## 9 Future perspectives and conclusion

The multifunctional role of NFE2L1 in regulating proteostasis, mitigating oxidative stress, and preserving neuronal health makes it a compelling therapeutic target for neurodegenerative diseases. Future research should focus on developing strategies to selectively activate NFE2L1 in neurons while minimizing systemic effects. Approaches such as small molecules, gene-editing tools, and compounds promoting its nuclear translocation and transcriptional activity hold promise for restoring proteostasis and combating neurodegenerative progression.

An exciting avenue for exploration lies in NFE2L1’s interplay with ferroptosis, particularly its regulation of antioxidant defenses and lipid metabolism. Investigating how NFE2L1 mitigates ferroptosis-driven damage could provide novel therapeutic strategies for diseases characterized by oxidative stress and lipid peroxidation. Furthermore, leveraging emerging tools like single-cell RNA sequencing, proteomics, and computational modeling could offer deeper insights into NFE2L1’s regulatory networks, enabling more precise targeting of vulnerable neuronal populations.

NFE2L1 also shows potential as a diagnostic biomarker, as reduced expression has been observed in affected brain regions of patients with AD and PD. Longitudinal studies examining NFE2L1 expression and activity in neurodegenerative disease progression could provide valuable insights into its predictive utility. The integration of these findings into personalized medicine approaches could pave the way for precision therapies tailored to individual patient profiles.

In conclusion, NFE2L1 serves as a master regulator of neuronal homeostasis, bridging critical pathways such as the UPS, autophagy, and ferroptosis to maintain cellular balance under stress. Its dysfunction contributes significantly to neurodegenerative disease progression, highlighting the need for innovative therapeutic strategies targeting this critical transcription factor. By addressing the existing knowledge gaps and leveraging advanced technologies, NFE2L1-based interventions hold the potential to revolutionize the treatment of neurodegenerative diseases, improving outcomes and quality of life for affected individuals.
